# Therapeutic prediction of HIV-1 DNA decay: a multicenter longitudinal cohort study

**DOI:** 10.1186/s12879-021-06267-5

**Published:** 2021-06-22

**Authors:** Yongsong Yue, Yijia Li, Yizhi Cui, Nidan Wang, Yunda Huang, Wei Cao, Yang Han, Ting Zhu, Wei Lyu, Jing Xie, Xiaojing Song, Yanling Li, Tong Wang, Tuofu Zhu, Taisheng Li

**Affiliations:** 1 Department of Infectious Diseases, Peking Union Medical College Hospital, Chinese Academy of Medical Sciences & Peking Union Medical College, No. 1 Shuaifuyuan, Wangfujing Street, Beijing, 100730 China; 2 Center for AIDS Research, Chinese Academy of Medical Sciences & Peking Union Medical College, Beijing, China; 3 Department of Infectious Diseases and Clinical Microbiology, Beijing Chao-yang Hospital, Capital Medical University, Beijing, China; 4 Division of Infectious Diseases, Massachusetts General Hospital and Brigham and Women’s Hospital, Harvard Medical School, Boston, MA USA; 5 Key Laboratory of Functional Protein Research of Guangdong Higher Education Institutes, Institute of Life and Health Engineering, College of Life Science and Technology, Jinan University, Guangzhou, 510632 China; 6 Vaccine and Infectious Disease Division, Fred Hutchinson Cancer Research Center, Seattle, WA USA; 7 Department of Global Health, University of Washington, Seattle, WA USA; 8 Department of Laboratory Medicine, School of Medicine, University of Washington, 325 Ninth Ave, Seattle, WA 98104-2499 USA

**Keywords:** HIV-1 DNA, Prediction model, Chronic infection, Combined antiretroviral therapy, CD4

## Abstract

**Background:**

Factors predicting peripheral blood total HIV-1 DNA size in chronically infected patients with successfully suppressed viremia remain unclear. Prognostic power of such factors are of clinical significance for making clinical decisions.

**Methods:**

Two sets of study populations were included: 490 China AIDS Clinical Trial (CACT) participants (Training cohort, followed up for 144 to 288 weeks) and 117 outpatients from Peking Union Medical College Hospital (PUMCH) (Validation cohort, followed up for more than 96 weeks). All patients were chronically HIV-1-infected and achieved successful HIV-1 plasma RNA suppression within week 48. Total HIV-1 DNA in blood at baseline, 12, 24, 48, 96, 144 and 288 weeks after combined antiretroviral therapy (cART) initiation were quantified. Generalized estimating equations and logistic regression methods were used to derive and validate a predictive model of total HIV-1 DNA after 96 weeks of cART.

**Results:**

The total HIV-1 DNA rapidly decreased from baseline [median = 3.00 log_10_ copies/10^6^ peripheral blood mononuclear cells (PBMCs)] to week 24 (median = 2.55 log_10_ copies/10^6^ PBMCs), and leveled off afterwards. Of the 490 patients who had successful HIV-1 plasma RNA suppression by 96 w post-cART, 92 (18.8%) had a low total HIV-1 DNA count (< 100 copies/10^6^ PBMCs) at week 96. In the predictive model, lower baseline total HIV-1 DNA [risk ratio (RR) = 0.08, per 1 log_10_ copies/10^6^ PBMCs, *P* < 0.001] and higher baseline CD4+ T cell count (RR = 1.72, per 100 cells/μL, *P* < 0.001) were significantly associated with a low total HIV-1 DNA count at week 96. In an independent cohort of 117 patients, this model achieved a sensitivity of 75.00% and specificity of 69.52%.

**Conclusions:**

Baseline total HIV-1 DNA and CD4+ T cell count are two independent predictors of total HIV-1 DNA after treatment. The derived model based on these two baseline factors provides a useful prognostic tool in predicting HIV-1 DNA reservoir control during cART.

**Supplementary Information:**

The online version contains supplementary material available at 10.1186/s12879-021-06267-5.

## Introduction

A critical goal of antiretroviral therapy is to reduce the size of the total HIV-1 DNA reservoir which poses a major obstacle for HIV-1 eradication [1–3]. Total HIV-1 DNA load has been associated with disease progression, treatment efficacy, and HIV-1 co-morbidities such as HIV-1-associated dementia and malignancy [4–7]. High total HIV-1 DNA levels during cART have been associated with faster viral rebound after structured treatment interruption (STI) [8–10], while low total HIV-1 DNA levels may increase the possibility of prolonged viral remission after cART interruption, which was observed in the Mississippi baby [11] and post-treatment controllers (PTCs) [12, 13]. In the VISCONTI cohort study, the median total HIV-1 DNA level was near 100 copies/10^6^ PBMCs in the 14 PTCs when cART was discontinued, which served as a milestone of relatively successful HIV-1 reservoir control, potentially leading to prolonged remission or functional cure [12].

It is generally accepted that early treatment is critical for the viral suppression of PTCs [12]. Numerous studies have investigated factors related to the dynamics of total HIV-1 DNA decay during cART, including treatment initiation time, antiretroviral therapy intensity, demographic data, baseline HIV-1 plasma RNA and DNA, CD4+ and CD8+ cell count and CD4/CD8 ratio. Early initiation of cART during primary infection, low baseline total HIV-1 DNA and high CD4+ nadir were shown to increase the likelihood of achieving low total HIV-1 DNA outcome [14–17]. In addition, a cross-sectional study has reported that 28% out of 522 patients who are initiated cART during the chronic phase can achieve low total HIV-1 DNA (< 150 copies/10^6^ PBMCs) after long-term viral suppression [18]. Thus, as it is associated with ART-free remission, total HIV-1 DNA could serve as a usefully virologic parameter for monitoring therapeutic effects.

For clinicians, to translate the HIV-1 DNA test result into a prognostic prediction of certain ART is intriguing, especially when the HIV-1 viral load remains undetectable. Our previous study has shown that after 96 weeks’ ART, low HIV-1 DNA level is significantly associated with higher CD4/CD8 ratio [19]. However, baseline factors that predict low total HIV-1 DNA level in chronic patients after treatment remain elusive.

As such, in this study, we included 490 chronically HIV-1-infected treatment-naïve patients enrolled in multicenter HIV-1 cohort studies [20–22] and 117 outpatients in China. For the first time, a statistical model predicting the possibility of achieving a low total HIV-1 DNA outcome (< 100 copies/10^6^ PBMCs) for patients with chronic HIV-1 infection under suppressive cART was derived and validated in two independent cohorts.

## Methods

### Subjects

The Institutional Review Board of the Peking Union Medical College Hospital approved this study, and written informed consents were obtained from all the study participants. All methods were performed in accordance with the relevant guidelines and regulations. This study consists of two sets of study populations: 490 China AIDS Clinical Trial (CACT) participants and 117 outpatients from Peking Union Medical College Hospital (PUMCH).

The 490 CACT participants were included from two previously established multicenter cohorts, Cohort-2009 (CACT0810, ClinicalTrials.gov, identifier NCT00872417, recruited in 2009, followed up for 288 weeks) [20, 21] and Cohort-2012 (CACT1215, ClinicalTrials.gov, identifier NCT01844297, recruited in 2012, followed up for 144 weeks) [22]. Those 490 patients were selected as the training cohort for developing the predictive model of total HIV-1 DNA. The inclusion criteria of the 490 participants were: (1) chronically HIV-1-infected adult; (2) treatment-naïve; (3) CD4 cell count ≤350 cells/μL in the Cohort-2009 and ≤ 500 cells/μL in the Cohort-2012. All subjects received a standard treatment regimen containing two types of nucleoside and nucleotide reverse transcriptase inhibitors and one non-nucleoside reverse transcriptase inhibitor [20–22]. The criteria for successful HIV-1 plasma RNA suppression: (1) HIV-1 plasma RNA were suppressed to < 50 copies/mL within 48 weeks post initiation of cART, (2) transient viremia with < 200 copies/mL at no more than one time-point during cART was allowed.

In addition, another cohort consists of 117 HIV-1-infected adult outpatients, who were successfully treated (treatment was the same as CACT patients) between 2009 to 2016 at PUMCH and followed up for more than 96 weeks, was used as an independent validation cohort. The inclusion criteria for both the training and validation cohorts were: (1) complete baseline characteristics including age, gender, transmission route, and subtype, CD4+ and CD8+ T cell count, and HIV-1 plasma RNA levels; (2) total HIV-1 DNA levels must be available at baseline and week 96, while for other time points of 12 w, 24 w, and 48 w, only less than 2 missing values were allowed; (3) baseline total HIV-1 DNA level ≥ 100 copies/10^6^ PBMCs; and (4) Successful HIV-1 plasma RNA suppression within week 48.

### Total HIV-1 DNA quantitation

Total HIV-1 DNA was extracted from peripheral blood containing 0.25–1 million PBMCs using the Qiagen QIAsymphony DNA Mini Kit (QIAGEN, Hilden, Germany). The SUPBIO total HIV-1 DNA Quantitative PCR Kit (SUPBIO, Guangzhou, China) was used for simultaneously quantitating total HIV-1 DNA and cell number, following the manufacturer’s instructions. The linear quantification range of the SUPBIO total HIV-1 DNA quantitative kit was 20 copies/10^6^ PBMCs to 100,000 copies/10^6^ PBMCs.

### Statistical methods

Total HIV-1 DNA and RNA values were log-transformed in all inferential analyses. Generalized linear models of the longitudinal data using a generalized estimating equation (GEE) were performed to select the factors that predict the binary treatment outcome—total HIV-1 DNA < 100 copies/10^6^ PBMCs at week 96. Logistic regression models were used to characterize how the binary treatment outcome can be predicted by the baseline values of factors identified as a significant predictor in the final GEE model. The receiver operating characteristic (ROC) curve was utilized to evaluate the performance of the prediction power of the logistic regression model in the training set. The software packages SPSS 22.0 (IBM Corporation, Armonk, New York, USA) and MATLAB (R2014a, MathWorks, Natick, MA) were used for data analyses. A *P* value of < 0.05 was considered statistically significant.

## Results

### Baseline characteristics

Baseline characteristics were summarized for both the training (*n* = 490) and validation (*n* = 117) cohorts in Table 1. For the training cohort, the majority of patients were aged 30–40 years, male (69.8%), infected via sexual transmission [men who have sex with men (MSM) 31.4%, heterosexual 52.7%], and infected by the CRF01_AE subtype (44.5%). The median CD4+ T cell count was 226 cells/μL and median CD4/CD8 ratio was 0.25. The median HIV-1 plasma RNA level was 4.66 log_10_ copies/mL, and median total HIV-1 DNA was 3.00 log_10_ copies/10^6^ PBMCs. For the validation cohort, the majority were male (92.3%) and 71.8% of them were MSM. However, the clinical characteristics, including CD4+ and CD8+ T cell counts and HIV DNA levels, were similar between the two cohorts.

**Table 1:** Patients’ characteristics at baseline

Characteristics	Training cohort (***n*** = 490)	Validation cohort (***n*** = 117)
Median age (years, IQR)	35 (28–44)	32 (28–43)
Gender (n, %)		
Male	342 (69.8)	108 (92.3)
Female	148 (30.2)	9 (7.7)
Transmission category (n, %)		
MSM	154 (31.4)	84 (71.8)
Heterosexual	258 (52.7)	18 (15.4)
Bisexual	14 (2.9)	4 (3.4)
Blood	7 (1.4)	0 (0)
Others/Unknown	57 (11.6)	11 (9.4)
Subtype (n, %)		NA
CRF01_AE	218 (44.5)	
C/CRF07_BC/CRF08_BC	123 (25.1)	
B/B′	55 (11.2)	
URF	18 (3.7)	
Unknown	76 (15.5)	
Median CD4 cell count (cells/μL, IQR)	226 (159–344)	189 (50–293)
Median CD8 cell count (cells/μL, IQR)	809 (590–1181)	627 (449–925)
Median CD4/CD8 ratio (IQR)	0.25 (0.18–0.40)	0.27 (0.13–0.45)
Median HIV-1 plasma RNA (log copies/mL, IQR)	4.66 (4.34–5.14)	4.48 (2.90–4.96)
Median total HIV-1 DNA (log copies/10^6^ PBMCs, IQR)	3.00 (2.66–3.33)	3.01 (2.74–3.29)

*Note*: *IQR* interquartile range, *MSM* men who have sex with men, *NA* not applicable, *CRF* circulating recombinant form, *PBMCs* peripheral blood mononuclear cells

### Total HIV-1 DNA decay during 288 weeks of cART

**Fig. 1:**
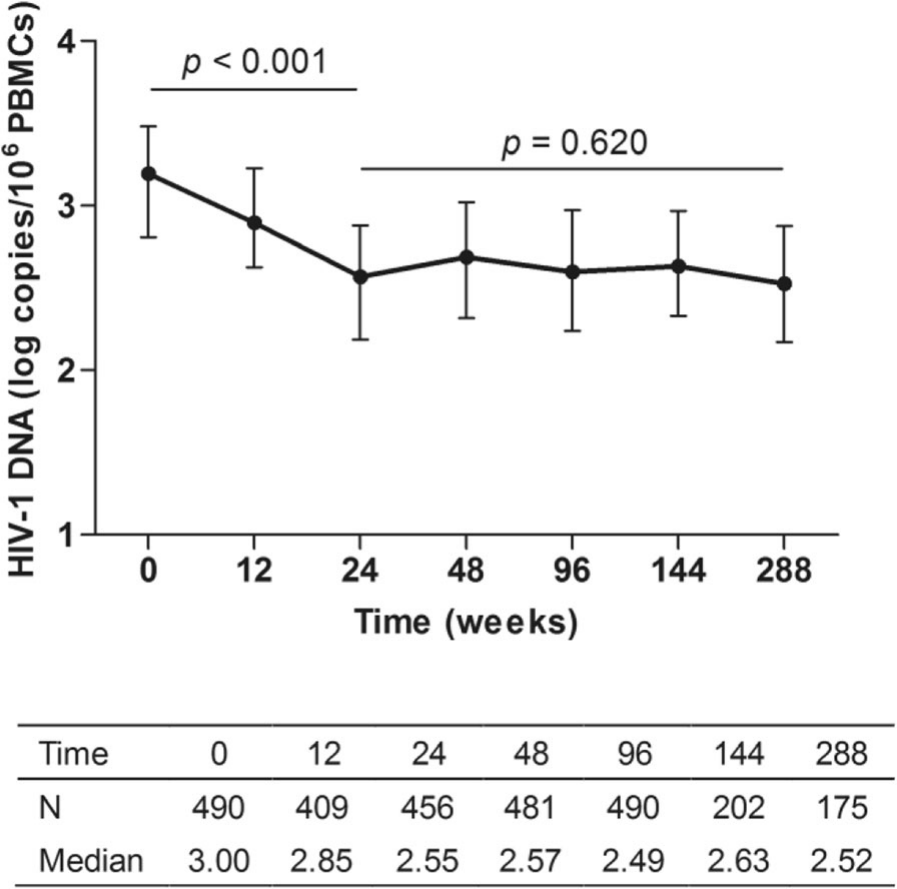
Decay of total HIV-1 DNA during 288 weeks of cART. N, Number of patients; Mean, the mean value of HIV-1 DNA at each follow-up time point; SD, standard deviation

During 288 weeks of cART, total HIV-1 DNA levels rapidly decreased from a median value of 3.00 log_10_ copies/10^6^ PBMCs at baseline to 2.55 log_10_ copies/10^6^ PBMCs at week 24 (*P* < 0.001, Fig. 1), then leveled off near the level of 2.55 log_10_ copies/10^6^ PBMCs during weeks 24 to 288 (*P* = 0.620, Fig. 1). In the following study, we consider the total HIV-1 DNA value at week 96, when all 490 patients had HIV-1 DNA data, as the HIV-1 DNA outcome after cART.

### Contribution of baseline factors to the total HIV-1 DNA decay

After 96 weeks of treatment, 18.8% (92/490) of subjects achieved total HIV-1 DNA levels of < 100 copies/10^6^ PBMCs. To identify which factors were associated with low HIV-1 DNA levels after 96 weeks of ART, univariate and multivariate logistic regression were used for the analysis. Univariate logistic regression revealed that a lower baseline total HIV-1 DNA level (RR = 0.07 per 1 log_10_ copies/10^6^ PBMCs, *P* < 0.001) and a higher baseline CD4+ T-cell count (RR = 2.11 per 100 cells/μL increase, *P* = 0.003) were beneficial factors associated with lower HIV-1 DNA levels at week 96 (Table 2). Consistently, in the multivariate logistic regression analysis, baseline factors predicting total HIV-1 DNA < 100 copies/10^6^ PBMCs at week 96 were also total HIV-1 DNA (RR = 0.08 per 1 log_10_ copies/10^6^ PBMCs, *P* < 0.001) and CD4+ T cell count (RR = 1.72 per 100 cells/μL increase, *P* < 0.001) (Table 2).

**Table 2:** Baseline factors associated with total HIV-1 DNA reduction to 100 copies/10^6^ PBMC level

Variables	Univariate Analysis		Multivariate Analysis	
	RR (95% CI)	***P***	RR (95% CI)	***P***
Sex		0.921		0.921
Male	1		1	
Female	0.96 (0.46–2.04)		0.96 (0.46–2.04)	
Age-per 10 years increase	1.02 (0.76–1.37)	0.891	1.02 (0.76–1.37)	0.889
Transmission route		0.359		0.305
MSM	1		1	
Heterosexual	1.06 (0.49–2.30)	0.885	1.15 (0.62–2.13)	0.650
Bisexual	0.24 (0.03–2.19)	0.203	0.27 (0.03–2.39)	0.241
Blood	1.33 (0.10–17.37)	0.826	0.85 (0.08–8.97)	0.895
Others/Unknown	0.35 (0.09–1.41)	0.140	0.35 (0.09–1.31)	0.120
Subtype		0.549		0.550
CRF01_AE	1		1	
C/CRF07_BC/CRF08_BC	1.47 (0.75–2.86)	0.264	1.47 (0.75–2.86)	0.263
B/B′	0.71 (0.23–2.21)	0.557	0.72 (0.23–2.22)	0.562
Others/Unknown	1.12 (0.31–4.01)	0.863	1.12 (0.31–4.00)	0.864
Baseline total HIV-1 DNA -per 1 log copies/10^6^ PBMCs	0.07 (0.03–0.16)	< 0.001	0.08 (0.04–0.16)	< 0.001
Baseline HIV-1 plasma RNA-per 1 log copies/mL	1.20 (0.74–1.96)	0.463	1.12 (0.70–1.77)	0.641
Baseline CD4 cell count-per 100 cells/μL	2.11 (1.28–3.48)	0.003	1.72 (1.35–2.20)	< 0.001
Baseline CD8 cell count-per 100 cells/μL	0.92 (0.81–1.05)	0.211	0.97 (0.90–1.04)	0.389
Baseline CD4/CD8 ratio-per 0.1 increase	0.87 (0.64–1.19)	0.391	0.86 (0.63–1.17)	0.340

*RR* relative risk, *CI* confidence interval, *MSM* men who have sex with men, *CRF* circulating recombinant form, *PBMCs* peripheral blood mononuclear cells

### Statistical model based on baseline DNA and CD4+ T cells to predict total HIV-1 DNA under cART

**Fig. 2:**
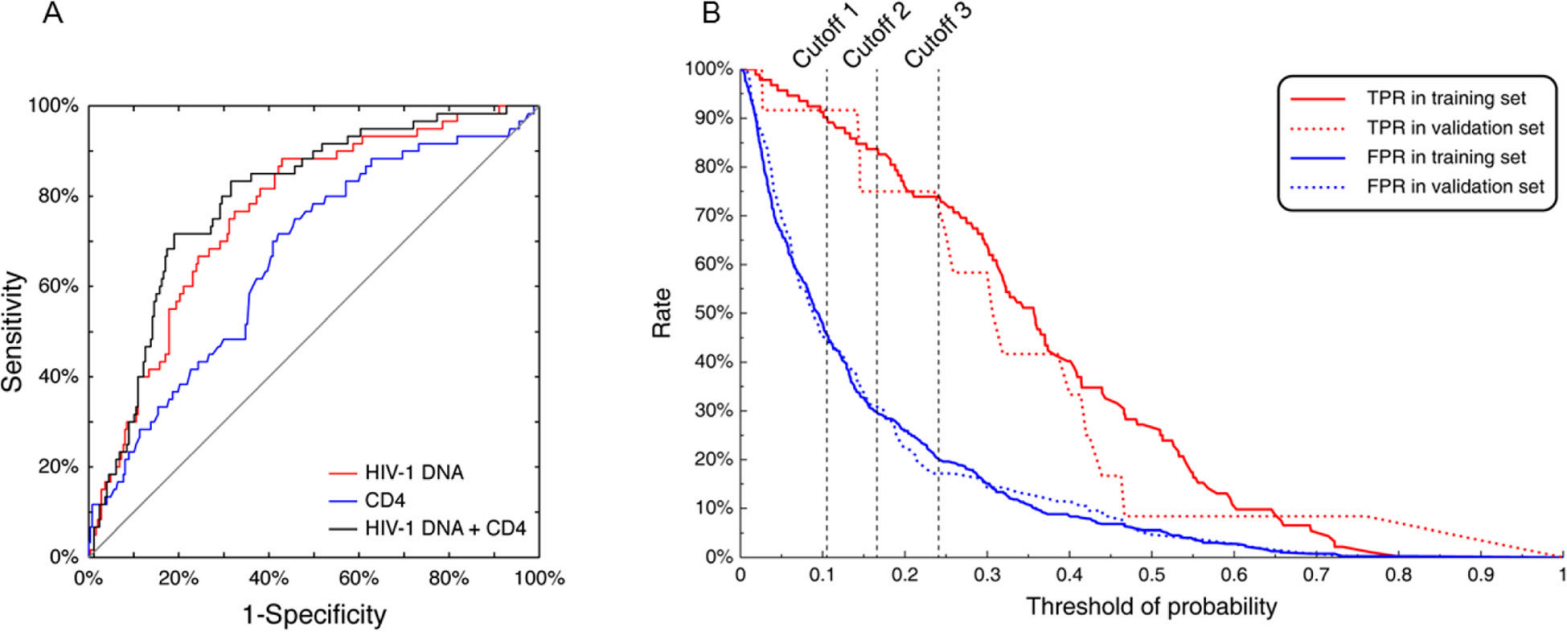
Total HIV-1 DNA prediction model. **A** ROC curves of different prediction models in the training cohort. **B** True positive rate (TPR, red) and False positive rate (FPR, blue) performance of the model in the training (solid lines) and the validation cohorts (dotted lines) with different cutoffs. Cutoff 1, the lowest threshold to obtain a TPR greater than 90%; Cutoff 2, the greatest TPR and FPR distance; Cutoff 3, the smallest threshold to obtain an FPR less than 20%

Further, generalized estimating equations (GEE) and logistic regression methods were used to derive a predictive model of total HIV-1 DNA < 100 copies/10^6^ PBMCs after 96 weeks of cART. With the GEE analysis, we found that longitudinal total HIV-1 DNA, CD4+ T cells, and CD4/CD8 ratio values were correlated with the total HIV-1 DNA outcome (Supplementary Table S1). Univariate and multivariate logistic regression analyses were then performed to relate the binary outcome with each identified predictor and combinations thereof. Total HIV-1 DNA and CD4 count were screened out as significant predictors of the treatment outcome of total HIV-1 DNA. Interestingly, the highest area under the ROC curve (AUC) of 0.82 was observed in the multivariate model with baseline total HIV-1 DNA and CD4 count other than each univariate model (Fig. 2A). As such, the best-performed predictive model based on baseline total HIV-1 DNA and CD4 count was derived:

logit(θ) = 4.579–2.518 × DNA + 0.477 × CD4 (Eq. 1).

Where θ represents the probability of achieving total HIV-1 DNA < 100 copies/10^6^ PBMCs at week 96 post-cART; DNA represents the baseline total HIV-1 DNA level in log_10_ copies/10^6^ PBMCs; and CD4 represents the baseline CD4+ T cell count in 100 cells/μL. For example, if a patient starts cART treatment with 300 (2.48 log_10_) copies/10^6^ PBMCs of baseline total HIV-1 DNA and a CD4 count of 500 cells/μL, there is a 72% (θ = 0.72) chance that his total HIV-1 DNA will fall below 100 copies/10^6^ PBMCs after 96 weeks of treatment according to eq. 1.

With Eq. 1, we validated the prediction model with the validation cohorts, which contained 117 outpatients from the real hospital settings. At cutoff 2, the greatest true positive rate (TPR) and false positive rate (FPR) distance, the predictive model performed similarly in both training and validation cohorts. TPRs were 83.70 and 75.00% while FTRs were 29.40 and 30.48% in the training and validation cohorts, respectively (Fig. 2B). In this independent cohort of 117 patients, this model achieved a sensitivity of 75.00% and specificity of 69.52%.

## Discussion

In this study, the characteristics and impact factors of HIV-1 DNA decay was studied with longitudinal cohorts. A probability model that predicts the outcome of HIV-1 DNA reservoir size after 2 years of cART using baseline values of total HIV-1 DNA and CD4+ T cell count was established for the first time. The model performed well and similarly in both the training cohort and an independent outpatient validation cohort, which indicated the robustness of the model.

Globally, most HIV-1 patients on treatment are chronically infected. Achieving remission or a potential functional cure in such patients is more challenging than in those who are acutely infected [10, 12]. Studies of the HIV-1 reservoir in chronic patients were limited by small populations or were cross-sectional [18, 23, 24]. To our knowledge, the data in the present study represent the largest longitudinal cohort study focusing on chronically HIV-1-infected individuals. We found that for chronically HIV-1-infected patients, total HIV-1 DNA decay after initiation of cART demonstrated a biphasic reduction within 96 weeks. Most decline happened in the first 6 months and then began to plateau, which was consistent with other findings in chronically HIV-1-infected patients [25, 26]. Compared with the studies by Besson et al. [25] and Gandhi et al. [27], our sample size was larger, and we detected the HIV-1 DNA at week 12 and 24. We suggested for the first time that the HIV-1 DNA decline was mainly within 24 weeks rather than 48 weeks (1 year). In addition, Gandhi et al. found that there was a positive correlation between the level of HIV-1 DNA before treatment and on treatment. Our study further confirmed that baseline HIV-1 DNA was an independent predictive factor of HIV-1 DNA after 2 years of treatment. Furthermore, 18.8% of chronic patients achieved < 100 total HIV-1 DNA copies/10^6^ PBMCs after 96 weeks of cART. In comparison, with prolonged treatment as long as the median duration of 13 years, it was reported that 28% of such patients could achieve ~ 150 total HIV-1 DNA copies/10^6^ PBMCs [18].

Our data showed that the pre-treatment total HIV-1 DNA burden and CD4+ T cell count correlate to long term total HIV-1 DNA control in real hospital settings. This model could be applicable for clinical decision-making on therapy strategies. Our findings suggest that chronically infected patients initiating cART with lower total HIV-1 DNA and higher CD4+ T cell baseline levels have a higher possibility of achieving low total HIV-1 DNA level with effective cART. In 2016, HIV-1 DNA has been used as one of the criteria for screening subjects in treatment-interruption study by Calin et al. [28]. In future studies such as treatment-interruption, de-escalation therapy, or cell or gene therapy, clinicians could select suitable subjects who may benefit more by preliminary prediction of HIV-1 DNA outcome prior to patients’ initial treatment. This study also supports initiatives for early treatment. Although delayed treatments can also keep viral plasma RNA within the detection limits, they maintain high DNA levels.

One limitation existed in our study was that we did not account for other possible mechanisms contributing to total HIV-1 DNA decay, including duration of infection, antibody responses [29], and polyfunctionality of HIV-1-specific T cells [30]. These present interesting directions for future hypothesis-driven investigations. In addition, the predictive model was established based on specific HIV-1 DNA testing methods and specific populations. The results of the study necessitate further verification in different settings and with different methods.

## Conclusions

In summary, this is a longitudinal study in a large population of chronically HIV-1-infected patients to evaluate the predictive factors associated with total HIV-1 DNA decay after cART. Based on baseline total HIV-1 DNA and CD4, we established a predictive model for treatment-naïve patients to estimate their likelihood of achieving total HIV-1 DNA < 100 copies/10^6^ PBMCs after suppressive cART. This model will be applicable for identifying potential cART-cessation candidates who are relatively more likely to achieve functional cure before cART initiation.

## Supplementary Information

**Additional file 1: TableS1.** Generalized Estimating Equation results of single factor/covariate.

## Data Availability

The datasets used and/or analyzed in the current study are available from the corresponding author on reasonable request.

## Abbreviations

cART: Combined antiretroviral therapy
PBMCs: Peripheral blood mononuclear cells
RR: Risk ratio
STI: Structured treatment interruption
PTCs: Post-treatment controllers
CACT: China AIDS Clinical Trial
PUMCH: Peking Union Medical College Hospital
GEE: Generalized estimating equation
ROC: Receiver operating characteristic
MSM: Men who have sex with men
AUC: Area under the ROC curve
TPR: True positive rate
FPR: False positive rate
IQR: Interquartile range
NA: Not applicable
CRF: Circulating recombinant form
CI: Confidence interval
