# Supplementary material for: Therapeutic prediction of HIV-1 DNA decay: a multicenter longitudinal cohort study
Source: BMC Infect Dis. 2021 Jun 22;21:592. doi: 10.1186/s12879-021-06267-5 (PMC8218450; doi:10.1186/s12879-021-06267-5)
Supplement: Supplementary file 1 — Additional file 1: TableS1. Generalized Estimating Equation results of single factor/covariate. [file 12879_2021_6267_MOESM1_ESM.docx]

# Supplemental Table 1: Generalized Estimating Equation results of single factor/covariate.

| **Model**  **Number** | **Factor/**  **Covariate** | **Case Processing Summary** | | | **Goodness of Fit** | | **Tests of Model Effects** | | **Parameter Estimates** | | | | |
| --- | --- | --- | --- | --- | --- | --- | --- | --- | --- | --- | --- | --- | --- |
|  |  | **N** | **Included** | **Excluded** | **QIC** | **QICC** | **Source** | **Sig.** | **Parameter** | **B** | **SE** | **Sig.** | **Exp(B)** |
| 1 | Subtype | 1535 | 1330 | 205 | 1349 | 1317 | (Intercept) | 0.000 | (Intercept) | -0.981 | 0.677 | 0.147 | 0.375 |
|  |  |  |  |  |  |  | Subtype | 0.272 | [Subtype=1] | -0.590 | 0.718 | 0.412 | 0.554 |
|  |  |  |  |  |  |  |  |  | [Subtype=2] | -0.073 | 0.719 | 0.919 | 0.929 |
|  |  |  |  |  |  |  |  |  | [Subtype=3] | -0.865 | 0.807 | 0.284 | 0.421 |
|  |  |  |  |  |  |  |  |  | [Subtype=4] | 0.000 | . | . | 1.000 |
| 2 | Gender | 1535 | 1535 | 0 | 1534 | 1518 | (Intercept) | 0.000 | (Intercept) | -1.235 | 0.284 | 0.000 | 0.291 |
|  |  |  |  |  |  |  | Gender | 0.469 | [Gender=1] | -0.239 | 0.330 | 0.469 | 0.788 |
|  |  |  |  |  |  |  |  |  | [Gender=2] | 0.000 | . | . | 1.000 |
| 3 | Route | 1535 | 1535 | 0 | 1508 | 1468 | (Intercept) | 0.000 | (Intercept) | -2.793 | 0.595 | 0.000 | 0.061 |
|  |  |  |  |  |  |  | Route | 0.086 | [Route=1] | 1.607 | 0.632 | 0.011 | 4.986 |
|  |  |  |  |  |  |  |  |  | [Route=2] | 1.597 | 0.636 | 0.012 | 4.938 |
|  |  |  |  |  |  |  |  |  | [Route=3] | 0.308 | 1.199 | 0.797 | 1.361 |
|  |  |  |  |  |  |  |  |  | [Route=4] | 1.184 | 1.247 | 0.342 | 3.267 |
|  |  |  |  |  |  |  |  |  | [Route=5] | 0.000 | . | . | 1.000 |
| 4 | Age | 1535 | 1525 | 10 | 1527 | 1510 | (Intercept) | 0.183 | (Intercept) | -0.796 | 0.597 | 0.183 | 0.451 |
|  |  |  |  |  |  |  | Age | 0.299 | Age | -0.018 | 0.017 | 0.299 | 0.983 |
| 5 | Week | 1535 | 1535 | 0 | 1527 | 1521 | (Intercept) | 0.000 | (Intercept) | -1.415 | 0.144 | 0.000 | 0.243 |
|  |  |  |  |  |  |  | Week | . | Week | 0.000 | 0.000 | 0.000 | 1.000 |
| 6 | VL | 1535 | 1412 | 123 | 1390 | 1385 | (Intercept) | 0.000 | (Intercept) | -1.420 | 0.144 | 0.000 | 0.242 |
|  |  |  |  |  |  |  | VL | 0.361 | VL | 0.000 | 0.001 | 0.361 | 1.000 |
| 7 | CD4 | 1535 | 1231 | 304 | 1255 | 1251 | (Intercept) | 0.000 | (Intercept) | -1.434 | 0.144 | 0.000 | 0.238 |
|  |  |  |  |  |  |  | CD4 | 0.002 | CD4 | 0.006 | 0.002 | 0.002 | 1.006 |
| 8 | CD8 | 1535 | 1224 | 311 | 1245 | 1241 | (Intercept) | 0.000 | (Intercept) | -1.413 | 0.144 | 0.000 | 0.243 |
|  |  |  |  |  |  |  | CD8 | 0.874 | CD8 | 0.000 | 0.001 | 0.874 | 1.000 |
| 9 | Ratio | 1535 | 1223 | 312 | 1244 | 1240 | (Intercept) | 0.000 | (Intercept) | -1.423 | 0.144 | 0.000 | 0.241 |
|  |  |  |  |  |  |  | Ratio | 0.046 | Ratio | 0.023 | 0.011 | 0.046 | 1.023 |
| 10 | DNA | 1535 | 1129 | 406 | 1105 | 1103 | (Intercept) | 0.000 | (Intercept) | -1.373 | 0.143 | 0.000 | 0.253 |
|  |  |  |  |  |  |  | DNA | 0.000 | DNA | -0.017 | 0.003 | 0.000 | 0.983 |

Note: QIC, Quasi-likelihood under Independence Model Criterion; QICC, Corrected Quasi-likelihood under Independence Model Criterion.

Gender: 1, male; 2, female.

Route: 1, MSM; 2, Heterosexual; 3, Bisexual; 4, Blood; 5, Unknown/others.

Subtype: 1, CRF01_AE; 2, CRF07/08BC and C; 3, B/B'; 4, URF.

VL: viral load; RNA: HIV-1 RNA; CD4: CD4+ T cell; CD8: CD8+ T cell; DNA: HIV-1 DNA.
